# Epidemic Erysipelas in Williamsville, Erie County, N. Y.

**Published:** 1846-02-01

**Authors:** WM. Van Pelt


### BUFFALO MEDICAL JOURNAL

**Vol. 1.** FEBRUARY 1, 1846. **No. 9.**

### Epidemic Erysipelas in Williamsville, Erie County, N. Y.

Communicated for the Buffalo Medical Journal, by WM. VAN PELT, M. D.

Epidemic Erysipelas appeared first in this place, Nov. 10th, 1842, and was the prevailing disease for the following four months. Since that period a few cases have occurred, at intervals, up to the 15th of March, 1845. The profession have been somewhat dilatory in naming this affection. The people call it the *"Black Tongue."* I never have seen a case in which the tongue was involved in the inflammation. No name yet given to it expresses fully its character. It manifested itself upon the skin generally in severe cases, but in mild attacks the lining membrane of the throat was the first point of accession. In a few cases the mucus and serous membranes of the abdomen suffered first. It sometimes changed its location as circumstances invited it. The uniformity of the symptoms in the cases which came under my notice was remarkable, excluding the symptoms peculiar to different organs, as they became the seat of this peculiar inflammation, which in most instances resembled acute inflammation of the parts. The first stage was ushered in by severe chills, as in an intermittent, attended by great agitation, nausea, and vomiting, and pain in the epigastrium in many instances. This latter symptom was by no means constant. Reaction gradually took the place of the chill; and lameness of the muscles, pain in the large joints, headache, pain in the back, sharp darting pain along the extremeties supervened. The organ in which the inflammation subsequently appeared, was always particularly noticed by the patient in the detail of symptoms. In a great majority of cases, the tonsils and mucus membrane exhibited an unusual redness, wtthout much tumefaction. This was the mildest form of the disease.—In a few cases the tonsils were so enlarged as to render deglutition impossible, and threaten suffocation; then, the other glands about the neck participated in the irritation. The pulse seldom numbered over 112 during the first 24 hours; it was hard and bounding, or unusually slow, full, and strong, increased a few pulsations upon standing up. Position did not seem to have much effect upon the more active pulse. The tongue was covered with thick white fur, and slimy mucus, possessing but little the appearance of healthy saliva. The cases of gastric irritation had the shining red tongue, and a scanty secretion from the glands of the mouth. The efflorescence usually appeared upon the surface in about 48 hours from the beginning of the attack, if it did at all. When it did appear, it spread from some gland which previously had been much swollen, exhibiting the characteristics of ordinary erysipelatous inflammation. The erysipelas of the head seldom extended upon the body. It did in three cases, passing over the entire surface in about eight weeks. One of them recovered. In four instances, the patients while suffering the primary symptoms, accidentally scratched themselves with a pin, and the inflammation spread from the wound. In one case it spread from a cut on the foot, in another from the finger nail, which had been slightly injured in cleaning, and in one, it was excited by a blister.

When the glands of the axilla were the first to take on the inflammation, and the efflorescence spread from them, it invariably passed down the arm to the hand, following the large veins, then spreading laterally around the limb. If it spread from the groin it passed down the leg in the same order. If the cutaneous disease did not make its appearance, the glands suppurated extensively.

As the erysipelas appeared upon the surface, the fever assumed a more serious character, prostration was more marked, the pulse rose to 125 or 130, and was small, soft and quick, the tongue soon became covered with a dark, brown fur, sometimes rough and cracked, and sordes accumulated upon the teeth. There were present great fatigue, mild delirium, sliding down in the bed, sleeping with the eyes half open, respiration quick, deglutition performed with difficulty, and fluids could be distinctly heard as they fell into the stomach. The inflammation, as it spread, became of a darker color, and the line bounding it abrupt—more so than when the symptoms were not quite so grave. When the tumefaction preceded the redness, the line was not so distinct, nor the vesication so apt to take place, the cuticle falling in scales.

When the mucus membrane of the Lungs was attacked, a dry, ringing cough, and general soreness of the chest, often involving the pleura, were added to the initiatory symptoms, bearing the character of Bronchitis, Pleuro-Pneumonia, or Typhoid-Pneumonia.

If the mucus membrane of the intestines, muco-enteritis, or dysentery followed.

A large number of cases of colic occurred, preceded by constipation, and, I believe, in every instance followed the same general, incipient symptoms. The spasm came on in the cold stage, was followed by symptoms of peritoneal inflammation, proving fatal in two instances, notwithstanding the obstruction was soon overcome.

The glands seldom suppurated, though, under other circumstances, extensive suppuration would be expected to follow so violent an inflammation; but the tumefaction subsided in a remarkably short time as the efflorescence spread upon the surface.

When the cellular tissue became involved, the adipose substance seemed to dissolve, the muscles were as cleanly dissected as could be done with a knife, and what remained of the cellular tissue, had much the appearance of wet tow.

Not one in ten who had the chill, &c., slightly, had any erysipelatous affection. Children were exempt entirely. More females than males, (say 5 to 3,) were attacked. I thought I could observe a tendency in the fever to terminate at regular periods, by crisis, as is sometimes seen in other fevers.

Whatever may have been the cause which gave rise to this singular affection, it was, at all events, a general one. I think it is a well established fact, that endemical influences commonly referred to the atmosphere, occasion diseases to appear in remote situations, where there has been no communication of individuals to extend them, or simultaneously to develope them through a wide extent of country. Scarlatina, Cynanche, or Rubeola were not prevailing at the time, nor had they for nearly two years previous. I did not see any appearances to confirm the suspicion that there was an intimate relation between either of these affections and the prevailing one. Typhus Fever had preceded it a short time, and in many cases the typhoid symptoms were marked, leading me to suspect there might be some relation between the two.

The Prognosis, as in other disorders, was governed by the age, sex, and habits of the patient, and the organ and textures affected. It was for the most part favorable when the efflorescence was confined to the mucus membrane of the fauces, with but little general derangement.

When the skin became the seat,after a short but very active inflammatory stage, it passed, like Typhus, into a collapse, especially when penetrating to the cellular membrane beneath. If the pleura, or peritoneum were affected, the cases that were fatal, were so in the space of three or four days. The aspect of the disease was most revolting, when the cellular membrane was extensively diseased. A long season of suffering was sure to follow, which the youth and middle aged endured better than those advanced in life.

Of Puerperal-peritonitis but seven cases occurred. Three I attended during their confinements, having at the same time erysipelatous patients under treatment. The remaining cases had the assistance of no physician, nor of any one else who had the care of patients affected with the disease under consideration. I found, upon close inquiry, that they all, either a short time previously, or at the time of confinement, were suffering under the mildest form of the Epidemic, supposing it to be an ordinary cold. The contagiousness of this affection will probably be a disputed point, until the principle generating it, and several forms of disease, can be made cognizable to the senses. I possess no evidence that it is more contagious than other epidemics.

Those who chanced to have the care of individuals affected with the severest forms of the affection, though in constant fear, contracted it with no more severity than those who feared to go near the sick. On reviewing the history of the puerperal cases which came under my observation, the following query has presented itself: Is not this puerperal disease, the same prevailing affection invited to the substance of the uterus and peritoneum, modified according to its location, as in other organs.

The treatment was such as the symptoms indicated, upon general principles. Those with whom I advised, with one or two exceptions, hesitated to sanction several important steps, entering, more or less, into the popular fear against active measures, though acknowledging that appearances seemed to demand them. The propriety of Venesection was questioned, but I hesitate not to say that there was a time, when, if resorted to, it would have more power in assisting to overcome this disease than any other one remedy. *But that period was much earlier than*

*appearances often indicated.* In two cases in which I feared to resort to it, thinking it too late, spontaneous hæmorrhage was followed by immediate relief—Nature doing what I neglected.

Out of 23 cases of external inflammation, I resorted to Venesection in 11. It afforded no relief to three of this number, but in the remaining eight, it was afforded in a short time, checking the progress of the inflammation entirely in five instances. It was not adopted in the remainder of the 23 cases, as the symptoms did not appear to demand it. When practiced in a few of the cases in which there was no cutaneous affection, it was no less efficient. In the cases of puerperal peritonitis which came under my care, I used the lancet freely. In three of that number there was no relief; the others recovered. Two cases within my knowledge, treated upon Thompsonian principles, were fatal in four or five days.

I was called, in but few instances, before the cold stage had passed;—but if so, warm drinks and external heat, to invite reaction, were employed. When this was somewhat established, an emetic of Ipecacuanha, in conjunction with warmth externally, if it induced free perspiration, which it usually did in the mild attacks, gave as prompt relief as the other method of depletion. When there was no gastric irritation, Cal. and Ipecac, aa gr viii, followed by Castor Oil in three hours, producing free evacuation, was quite as effectual; but if the least abdominal or thoracic disease existed, the mildest laxatives were better, at the same time keeping up gentle diaphoresis by means of warm drinks, Dov. pulv., Ipecac, Camphor, &c.

As a tonic, in the stage of collapse, Sulphate of Quinine, with diffusible stimuli answered the indications in all cases involving congestion.—Sulph. Quinine and Capsicum, aa grs ii, taken every two hours, was a favorite prescription. Often these quantities were much increased, bringing a pulse which could not be counted, down to an 112 or 120.

The preparations of Opium were highly useful in the different stages.

As an application to the fauces, particularly when the parts assumed a dark color, Capsicum was preferred to any other. An infusion of Sage and Honey, when the redness was of a bright red color, answered that condition better.

Nitrate of Silver, as an external application, from its well known reputation in ordinary erysipelatous inflammation, I was induced to apply. I did so in every manner directed, both upon the sound and diseased surfaces, and it did not answer the expectations previously anticipated, proving, in fact, worse than useless. I can say the same of several powerful applications recommended by the highest authority, excepting those cases where the inflammation manifested a tendency to metastasis internally.

Mercurial Ointment, as strong as can be made, applied repeatedly upon those parts of the body where the skin is thinest, until its effects were observable in the mouth, proved a salutary remedy. Warm poultices, such as are commonly prescribed in acute local inflammations, were liable to produce much mischief, particularly when there was prostration, and I found as much consideration necessary in applying them as in the employment of venesection. A lotion of alcohol and water applied by means of saturated clothes, except in cases that were congestive, I found most useful in affording comfort to the patient. Of Incisions I cannot say much, as I employed them in but one instance. They afforded relief, discharging a sanious matter, and I have no doubt prevented extensive destruction of the cellular substance. An alcoholic solution of creasote, very weak, injected into the sinuses, excited healthy granulation, and corrected the odor of the discharges.

The success of the above treatment, the outlines of which I have imperfectly given, will warrant its correctness. I lost 3 in 24, from the severe cutaneous and cellular variety, and 2 in 7, of the puerperal.—The mildest applications I found to give the best satisfaction. The hope to overcome a disease like this by a local application, I believe to be perfectly futile. When the pathology of Erysipelatous Fever is better understood, this idea will be abandoned.

*Williamsville, Dec.* 9, 1845.

